# Confidence-aware pseudo-label selection and verifier training for semi-supervised LLM reasoning with minimal labels

**DOI:** 10.3389/frai.2026.1857934

**Published:** 2026-08-26

**Authors:** Keizo Kato, Chenhui Chu, Yugo Murawaki, Sadao Kurohashi

**Affiliations:** 1 Fujitsu Limited, Kawasaki, Japan; 2 Graduate School of Informatics, Kyoto University, Kyoto, Japan; 3 National Institute of Informatics, Tokyo, Japan

**Keywords:** confidence aware, entropy calibration, LLM, pseudo-label selection, reinforcement learning, semi-supervised learning

## Abstract

This paper studies how to improve the reasoning ability of large language models (LLMs) with minimal supervision. Recent gains in LLM reasoning largely come from learning intermediate reasoning traces, and many methods reduce supervision cost by using traces whose final answers are correct. In realistic settings, however, obtaining even those answer labels can be costly, motivating methods that extend reasoning from a very small labeled set with abundant unlabeled questions. Verifier-based semi-supervised learning is a promising approach: a verifier trained on a small labeled set can score reasoning traces on unlabeled questions and identify candidates for pseudo-labeling. However, even with a verifier, it remains unclear how pseudo-labeled samples should be selected to support downstream training. In particular, pseudo-label selection must balance quality and quantity. To address this, we introduce an adaptive threshold selection policy that chooses thresholds on validation data using pseudo-label precision and sample count. We further combine this policy with confidence-aware verifier training to support confidence-based selection. Experiments on verifiable math reasoning benchmarks show that, under our training setup, this combination improves downstream reasoning accuracy over the tested baselines and selects pseudo-labeled subsets with a more favorable reliability–coverage trade-off. These results suggest a practical design direction for verifier-guided pseudo-label selection in answer-verifiable, minimal-label reasoning settings.

## 1 Introduction

Large language models (LLMs) are becoming increasingly capable of handling complex problems, and a major driver of this progress has been the explicit use and learning of intermediate reasoning. In particular, methods such as Chain-of-Thought have shown that making multi-step reasoning explicit can substantially improve performance on challenging tasks (Wei et al., 2022; Wang et al., 2022; Yao et al., 2023a,b). Building on this line of work, recent studies have also explored reusing model-generated reasoning traces for self-improvement, often treating generated solutions with correct final answers as useful supervision signals (Zelikman et al., 2022; Shinn et al., 2023; Madaan et al., 2023; Xiang et al., 2025; Peng et al., 2025). Taken together, these studies suggest that reasoning ability can be improved without exhaustively annotating every intermediate step by hand.

However, even when intermediate reasoning itself need not be fully annotated, many such approaches still assume access to a sufficient amount of labeled final answers. In expert domains and emerging tasks, preparing even these answer labels can be costly. This raises a practical question that is still not well understood: how far can reasoning ability be extended when *answer supervision itself* is available only in a very limited amount?

To address this challenge, recent work has explored semi-supervised reasoning, where a small labeled set is combined with a large pool of unlabeled questions. A promising approach is verifier-based semi-supervised learning: a verifier is trained from a small number of labeled examples, then applied to reasoning traces generated for unlabeled questions, and only reliable traces are reused as pseudo-labels for downstream self-training (Kato et al., 2026b; Park and Caragea, 2024; Moradi et al., 2025). This framework is appealing because judging whether a generated reasoning trace is valid is often easier than generating a valid trace from scratch.

Nevertheless, in verifier-based semi-supervised reasoning, the central challenge is not merely to introduce a verifier, but to decide *which* pseudo-labeled samples should actually be retained. Even when a verifier assigns confidence scores to candidate reasoning traces, an inappropriate selection policy may admit noisy pseudo-labels, while overly conservative filtering may retain too few samples to realize the benefits of semi-supervised learning. In other words, pseudo-label selection is not simply a confidence-filtering step; it is an *operating-point design problem* that directly affects downstream self-training. The key question is therefore how to balance the *quality* and *quantity* of pseudo-labels.

This view is consistent with the broader semi-supervised learning literature. Pseudo-labeling is known to suffer from confirmation bias when incorrect pseudo-labels are repeatedly reused (Lee, 2013; Arazo et al., 2020). Fixed-threshold methods such as FixMatch retain only sufficiently confident samples (Sohn et al., 2020), while later approaches adapt the threshold or weighting rule to better manage the quantity–quality trade-off (Zhang et al., 2021; Wang Y. et al., 2023; Chen et al., 2023; Rodemann et al., 2023). At the same time, our setting extends conventional class-label pseudo-labeling in an important way: here the decision variable is verifier confidence over generated reasoning traces, and the final objective is not classification quality alone but the utility of the retained pseudo-labeled subset for downstream reasoning improvement. In this verifier-guided reasoning setting, threshold design itself has remained relatively underexplored.

From this perspective, entropy is a natural confidence signal for pseudo-label selection. For classifiers trained with standard cross-entropy, lower-entropy predictions are generally associated with higher confidence. Therefore, low-entropy predictions are often useful candidates for pseudo-labeling, but entropy alone does not solve the selection problem. The threshold directly determines the trade-off between pseudo-label precision and retained sample count, and thus the usefulness of the selected subset for downstream training. Pseudo-label selection is therefore not simply a matter of taking the most confident samples, but of choosing an operating point that best balances reliability and coverage under limited supervision. In this work, we make this design issue explicit. Instead of relying on a fixed top-*k* rule, we introduce an *adaptive threshold selection* policy that chooses the operating point on validation data by considering both pseudo-label precision and retained sample count.

At the same time, adaptive threshold selection can work well only if the verifier's confidence is itself reliable. Entropy is often a useful confidence signal, but depending on how the verifier is trained, its correspondence with correctness can weaken. In particular, reinforcement learning objectives that improve prediction performance can also make the verifier overconfident on incorrect predictions (Kato et al., 2026a; Bereket and Leskovec, 2025). Conversely, training that merely sharpens confidence does not necessarily ensure correctness (Agarwal et al., 2025; Zhao et al., 2025; Li et al., 2025). This suggests that, in our setting, the verifier should not only be accurate as a correctness classifier, but should also produce confidence signals that are genuinely useful for sample selection. To this end, we build on Confidence-aware verifier training (CAVT) for improving the alignment between entropy and correctness (Kato et al., 2026a), and study how it interacts with adaptive pseudo-label selection in minimal-label semi-supervised reasoning.

These observations motivate us to examine the interaction between pseudo-label selection and verifier confidence training in the answer-verifiable, minimal-label setting considered in this work. Based on this view, this paper makes the following contributions: (1) we frame pseudo-label selection in verifier-based semi-supervised reasoning as a *quality–quantity operating-point problem* under minimal supervision; (2) we propose a practical adaptive threshold-selection policy that chooses pseudo-label operating points from validation data rather than relying on a fixed threshold; and (3) we combine this policy with confidence-aware verifier training and empirically show that, in our evaluated setting, the combination improves downstream reasoning performance relative to the tested baselines.

## 2 Related work

### 2.1 Reasoning self-training and verifier-based semi-supervised learning

A large body of work has shown that making intermediate reasoning explicit can substantially improve LLM performance, including Chain-of-Thought, Self-Consistency, Tree-of-Thoughts, and ReAct (Wei et al., 2022; Wang et al., 2022; Yao et al., 2023a,b). Building on this line, several studies have reused model-generated reasoning traces for self-improvement, such as STaR, Reflexion, Self-Refine, Meta-CoT, and ReGenesis (Zelikman et al., 2022; Shinn et al., 2023; Madaan et al., 2023; Xiang et al., 2025; Peng et al., 2025). A common practical assumption in this line of work is that a generated trace whose final answer is correct can be treated as useful supervision, or at least as a high-value candidate for further filtering. This answer-level criterion is attractive because it is simple, scalable, and broadly applicable to verifiable domains where step-level annotations are unavailable.

Recent work has also introduced explicit verification modules for generated reasoning or outputs. Examples include process verification, auxiliary judging models, and collaborative verification frameworks (He et al., 2024; Hsu et al., 2024; Liang et al., 2024; Ma et al., 2025; Jang et al., 2025). Closer to our setting, prior work has explored semi-supervised learning with verifiers or auxiliary judges. Our earlier framework trains a lightweight verifier from minimal labels and uses it to filter pseudo-labeled reasoning traces for downstream self-training (Kato et al., 2026b). VerifyMatch similarly combines pseudo-labeling with a verifier-like mechanism in semi-supervised learning, and VDS-TTT uses a learned verifier to select high-confidence self-generated samples for adaptation (Park and Caragea, 2024; Moradi et al., 2025).

Our work follows this practically important verifier-guided setting and focuses on verifiers trained with answer-level correctness labels. We do not assume that answer-level correctness is a complete characterization of reasoning-trace quality. Rather, we study a widely used and operationally convenient regime in which final-answer correctness provides the available supervision signal for training a verifier, and the central question becomes how to use the verifier's confidence to select pseudo-labeled traces more effectively. Within this scope, our contribution is to show that pseudo-label selection should not be treated as a fixed filtering heuristic, but as an operating-point design problem that balances pseudo-label reliability and retained sample count.

However, while these studies show that verifiers can improve pseudo-label quality, they usually do not make the threshold-selection rule itself a central design problem. In many cases, verification acts as a quality-control component, while the final selection policy remains a fixed threshold or a simple heuristic. Our work focuses on this missing connection by treating pseudo-label selection itself as a first-class design problem in verifier-based semi-supervised reasoning.

### 2.2 Utility of imperfect, invalid, and negative reasoning traces

A complementary line of work suggests that the usefulness of reasoning traces cannot always be reduced to final-answer correctness. Invalid or imperfect Chain-of-Thought demonstrations can retain much of the benefit of standard CoT prompting, with factors such as relevance and correct ordering sometimes mattering more than strict demonstration validity (Wang B. et al., 2023). Related work further argues that the distributional fit of synthetic reasoning traces to the student model may matter as much as, or in some cases more than, their final correctness (Chandra et al., 2026). These findings suggest that a trace ending in an incorrect answer may still contain useful local reasoning structure, partial solution steps, or model-compatible trajectories.

Other studies examine how negative, mistaken, or corrected examples can be used constructively. Contrastive CoT uses positive and negative reasoning patterns to improve reasoning behavior (Chia et al., 2023). Negative-data distillation leverages incorrect or low-quality reasoning data as a source of training signal when it is properly structured (Li et al., 2023). Mistake-correction and mistake-tuning approaches likewise show that LLMs can benefit from examples of previous errors when the errors are labeled, contrasted, corrected, or used to elicit self-rethinking (An et al., 2024; Tong et al., 2024; Alazraki et al., 2025). At the same time, noisy rationales can also harm reasoning when they are irrelevant or inaccurate (Zhou et al., 2024). Thus, imperfect traces are not uniformly useful or harmful; their value depends on how the error is represented and how the training objective uses it.

This literature is closely related to, but distinct from, our present scope. Our method is designed for the common minimal-label setting in which the verifier is trained from final-answer correctness labels and then used to select positive pseudo-labeled traces for downstream SFT. Under this setting, final-answer correctness is not claimed to be the best possible proxy for trace quality; it is the supervision signal assumed by many scalable reasoning self-training and verifier-guided selection pipelines. Extending our framework to exploit rejected traces, final-wrong traces, contrastive negatives, or corrected reasoning traces is an important future direction. Such extensions would require additional design choices about how to label, mask, contrast, or repair imperfect traces, which are orthogonal to the main question studied here: how to choose a useful confidence-based operating point when the verifier itself is trained under answer-level supervision.

### 2.3 Pseudo-label selection and adaptive thresholding

Pseudo-labeling is well known to suffer from confirmation bias when incorrect pseudo-labels are repeatedly reused in training (Lee, 2013; Arazo et al., 2020). As a result, confidence-based sample selection has become a central component of semi-supervised learning. This issue is also related to informative missingness in semi-supervised learning, where the labeled or selected subset is not missing at random and the selection mechanism can affect the resulting estimator (Wu et al., 2026). From this perspective, confidence-based pseudo-label selection should be treated not only as a filtering heuristic, but also as a mechanism that shapes the distribution of training signals. Fixed-threshold methods such as FixMatch retain only sufficiently confident pseudo-labels (Sohn et al., 2020), while later approaches such as FlexMatch and FreeMatch adapt the threshold according to class difficulty or learning status (Zhang et al., 2021; Wang Y. et al., 2023). SoftMatch further frames pseudo-labeling as a quality–quantity trade-off and replaces hard thresholding with soft weighting (Chen et al., 2023). More broadly, pseudo-label selection has also been discussed as a decision problem, where the choice of retained samples should be guided by downstream utility rather than by ad hoc rules alone (Rodemann et al., 2023).

This perspective is closely related to the broader literature on quality–coverage trade-offs in selective prediction (El-Yaniv and Wiener, 2010). In addition, UPS emphasizes that even high-confidence pseudo-labels can be harmful when they are wrong, motivating confidence-aware selection (Rizve et al., 2021). These studies provide important background for our threshold-selection design. However, in verifier-based reasoning settings, relatively little work explicitly selects thresholds by jointly considering pseudo-label precision and retained sample count on validation data. Prior verifier-based reasoning work has shown the usefulness of entropy filtering (Kato et al., 2026b), but still relied on a fixed top-*k* policy. In contrast, our method formulates threshold selection as a validation-based utility maximization problem that explicitly balances pseudo-label quality and quantity.

### 2.4 Confidence estimation, calibration, and confidence-aware training

For confidence-based pseudo-label selection to work well, the confidence signal itself must remain informative. Entropy and maximum probability have long been used as lightweight confidence signals for neural models and language models (Hendrycks and Gimpel, 2017; Liang et al., 2018; Desai and Durrett, 2020; Kadavath et al., 2022). Post-hoc calibration methods such as temperature scaling and beta calibration aim to improve the reliability of predictive probabilities (Guo et al., 2017; Kull et al., 2017), and recent work has extended this direction to LLMs through adaptive temperature scaling, Thermometer, and related methods (Xie et al., 2024; Shen et al., 2024; Zhao et al., 2024). Other studies propose richer confidence measures such as semantic entropy and semantic-entropy probes (Farquhar et al., 2024; Kossen et al., 2024).

Recent work also suggests that trace-level signals may provide additional information beyond final-answer verifier confidence. Lexical uncertainty markers in reasoning chains can help predict incorrect responses and support lightweight post-hoc calibration (Vanhoyweghen et al., 2025). Reasoning-chain length and trajectory dynamics can also be non-trivially related to performance, with longer reasoning not necessarily implying better answers (Ballon et al., 2025, 2026). These findings point toward richer trace-level confidence features, such as uncertainty markers, length effects, and trajectory-level diagnostics. In this paper, we focus on entropy-based confidence from an answer-level verifier because it is lightweight and directly compatible with verifier-guided pseudo-label selection under minimal supervision. Incorporating richer trace-internal confidence signals is a promising future extension.

At the same time, reinforcement learning can improve reasoning accuracy while degrading the usefulness of entropy as a confidence signal. Correctness-oriented RL objectives such as GRPO may increase performance but also induce overconfident errors, weakening the entropy–correctness correspondence (Kato et al., 2026a; Bereket and Leskovec, 2025). Conversely, entropy-minimization-style RL can sharpen confidence without directly ensuring correctness (Agarwal et al., 2025; Zhao et al., 2025; Li et al., 2025). CAVT addresses this tension by encouraging low entropy when predictions are correct and discouraging it when they are incorrect (Kato et al., 2026a). In our setting, this is particularly relevant because adaptive thresholding depends on the verifier not only being accurate as a classifier, but also producing confidence scores that are useful for selecting high-utility pseudo-labels.

Overall, prior work has shown the promise of verifier-based semi-supervised reasoning, adaptive pseudo-label selection, confidence-aware training, and learning from imperfect reasoning traces. Our work focuses on the interaction between verifier confidence and threshold selection in the widely used answer-level correctness setting. In particular, little attention has been paid to how pseudo-label selection and verifier training should be jointly designed so that confidence is useful for retaining high-utility pseudo-labels. To address this gap, we propose a unified framework that combines validation-based pseudo-label selection with confidence-aware verifier training for semi-supervised LLM reasoning under minimal supervision.

## 3 Method

In this section, we build on an existing verifier-based semi-supervised reasoning framework for learning from minimal labels (Kato et al., 2026b) and develop our method by extending it in two complementary directions. First, we revisit pseudo-label selection and introduce a validation-based criterion that explicitly balances pseudo-label quality and quantity for downstream self-training. We then improve the usefulness of verifier confidence for this selection process by introducing confidence-aware training.

### 3.1 Verifier-based semi-supervised reasoning from minimal labels

We begin from a verifier-based semi-supervised reasoning framework for learning from minimal labeled data. Let $D_{\text{lab}}={\left\{\left(x_{i},y_{i}\right)\right\}}_{i=1}^{N_{\ell }}$ be a small labeled set of problems and gold answers, and let $D_{\text{unlab}}={\left\{x_{j}\right\}}_{j=1}^{N_{u}}$ be a large unlabeled set. We use a generator *M*_gen_ and a verifier *M*_ver_. For each labeled example (*x*_*i*_, *y*_*i*_) ∈ *D*_lab_, the generator produces a reasoning trace and an answer, (*r*_*i*_, ŷ_*i*_) = *M*_gen_(*x*_*i*_), from which a correctness label $z_{i}\in \left\{0,1\right\}\subset V$ is derived using the gold answer. This yields the verifier-training set in Equation 1. The verifier assigns a next-token distribution over the vocabulary V, as shown in Equation 2, and is trained by minimizing the cross-entropy objective in Equation 3:


$$\begin{array}{l} D_{\text{ver}}={\left\{\left(x_{i},r_{i},{ŷ}_{i},z_{i}\right)\right\}}_{i=1}^{N_{\ell }}, \end{array}$$ (1)

$$\begin{array}{l} p_{i}\left(v\right)=M_{\text{ver}}\left(v|x_{i},r_{i},{ŷ}_{i}\right),\;v\in V, \end{array}$$ (2)

$$\begin{array}{l} L_{\text{ver}}=-\underset{\left(x_{i},r_{i},{ŷ}_{i},z_{i}\right)\in D_{\text{ver}}}{\sum }\log p_{i}\left(z_{i}\right). \end{array}$$ (3)

After verifier training, the generator is applied to each unlabeled question *x*_*j*_ ∈ *D*_unlab_, producing (*r*_*j*_, ŷ_*j*_) = *M*_gen_(*x*_*j*_). The verifier then assigns a next-token distribution over the vocabulary for each generated candidate:


$$\begin{array}{l} p_{j}\left(v\right)=M_{\text{ver}}\left(v|x_{j},r_{j},{ŷ}_{j}\right),\;v\in V. \end{array}$$ (4)

We define the verifier's predicted binary label as


$$\begin{array}{l} {ẑ}_{j}=\arg\underset{z\in \left\{0,1\right\}}{\max}p_{j}\left(z\right), \end{array}$$ (5)

and define the positive pool as the unlabeled candidates for which ẑ_*j*_ = 1. Confidence is measured by the token entropy of the verifier's next-token distribution:


$$\begin{array}{l} H\left(p_{j}\right)=-\underset{v\in V}{\sum }p_{j}\left(v\right)\log p_{j}\left(v\right). \end{array}$$ (6)

We refer to this token entropy simply as entropy unless otherwise stated. In this formulation, correctness labels are represented as output tokens of the verifier. In our experiments, we use two label tokens corresponding to correct and incorrect solutions, but the formulation itself is not restricted to the binary case. The predicted label in Equation 5 is obtained by comparing the full-vocabulary probabilities assigned to the candidate label tokens. For confidence estimation, we use the entropy of the full next-token distribution in Equation 6, rather than normalizing the distribution over only the candidate label tokens. This treats confidence as the sharpness of the verifier's generative prediction and keeps the same selection rule applicable to verifier settings with more than two labels, different label words, or other discrete verification outputs.

Pseudo-labeled samples are selected from the entropy-ranked positive pool using a top-*k* threshold. Specifically, let $D_{\text{unlab}}^{+}=\left\{\left(x_{j},r_{j},{ŷ}_{j},p_{j}\right)|{ẑ}_{j}=1\right\}$ be the positive pool, ranked by increasing entropy *H*(*p*_*j*_). For a given retention rate *k*, let τ_*k*_ denote the entropy cutoff corresponding to the top *k%* of this ranked positive pool, and define the retained pseudo-labeled subset in Equation 7:


$$\begin{array}{l} D_{\text{pseudo}}^{\left(k\right)}=\left\{\left(x_{j},r_{j},{ŷ}_{j}\right)|\left(x_{j},r_{j},{ŷ}_{j},p_{j}\right)\in D_{\text{unlab}}^{+},\;H\left(p_{j}\right)<\tau_{k}\right\}. \end{array}$$ (7)

Prior verifier-based semi-supervised reasoning work used this top-*k* selection rule with a fixed value of *k*, so the operating point was pre-determined rather than selected from validation data.

The downstream training set is then formed by combining the original labeled set with the retained pseudo-labeled samples, as shown in Equation 8:


$$\begin{array}{l} D_{\text{train}}^{\left(k\right)}=D_{\text{lab}}\cup D_{\text{pseudo}}^{\left(k\right)}. \end{array}$$ (8)

Using $D_{\text{train}}^{\left(k\right)}$ in Equation 8, we fine-tune the downstream generator by standard supervised fine-tuning (SFT). Specifically, the downstream objective is defined in Equation 9 as


$$\begin{array}{l} L_{\text{SFT}}^{\left(k\right)}=-\underset{\left(x,y^{⋆}\right)\in D_{\text{train}}^{\left(k\right)}}{\sum }\log P_{M_{\text{gen}}}\left(y^{⋆}|x\right), \end{array}$$ (9)

where *y*^⋆^ denotes the target output used for downstream training: for labeled examples in *D*_lab_, *y*^⋆^ is the gold supervision, while for pseudo-labeled examples in $D_{\text{pseudo}}^{\left(k\right)}$, *y*^⋆^ is the retained model-generated solution consisting of the reasoning trace and final answer.

Together, Equations 1–9 define a simple semi-supervised pipeline in which the generator first produces candidate reasoning traces, the verifier is trained according to Equations 2, 3, the verifier then scores unlabeled candidates according to Equation 4, confidence is measured by the token entropy in Equation 6, pseudo-labeled samples are selected by the top-*k* rule in Equation 7, and the retained samples are reused for downstream SFT through Equations 8, 9. At the same time, these equations also expose two design questions. First, the selection rule in Equation 7 depends on the choice of *k*, which may not optimally balance pseudo-label quality and retained sample count across datasets and verifier behaviors. Second, although Equations 2, 3 train the verifier to predict correctness, this alone does not guarantee that the token entropy in Equation 6 will rank pseudo-label reliability well. These limitations motivate the two components of our method: adaptive threshold selection, described next, and confidence-aware verifier training, introduced in Section 3.3.

### 3.2 Adaptive threshold selection

In verifier-based semi-supervised learning, pseudo-label selection is not merely a matter of keeping the most confident samples. Rather, it requires balancing pseudo-label quality and retained sample count for downstream training. This trade-off is especially important under minimal-label conditions, where overly permissive filtering can introduce harmful noise, while overly conservative filtering can leave too little data for self-training to be effective. We therefore treat the choice of the top-*k* operating point in Equation 7 as a utility-based decision problem, consistent with prior views of pseudo-label selection and quality–coverage trade-offs in selective prediction (Rodemann et al., 2023; El-Yaniv and Wiener, 2010).

Accordingly, a practical thresholding rule should prefer reliable subsets, avoid thresholds that retain too few samples, and remain cautious when precision is estimated from only a small validation subset. For each candidate retention rate *k*, we evaluate the corresponding subset selected by the top-*k* rule in Equation 7 on the verifier validation set. Let $D_{\text{val}}^{+}$ be the subset of verifier-validation examples predicted as positive by the verifier, and let $N_{\text{val}}^{+}=|D_{\text{val}}^{+}|$. Using the same token-entropy-based ranking as in Equation 7, we consider the retained validation subset


$$D_{\text{pseudo},\text{val}}^{\left(k\right)}=\left\{\left(x_{j},r_{j},{ŷ}_{j},y_{j}\right)\in D_{\text{val}}^{+}|H\left(p_{j}\right)<\tau_{k}\right\},$$

where *y*_*j*_ is the gold answer for validation example *x*_*j*_. We define the retained sample count as $n\left(k\right)=|D_{\text{pseudo},\text{val}}^{\left(k\right)}|$, and the retained positive-pool ratio as


$$\begin{array}{l} \rho \left(k\right)=\frac{n\left(k\right)}{N_{\text{val}}^{+}}. \end{array}$$ (10)

We then define the observed precision at retention rate *k* as


$$\begin{array}{l} \text{Prec}\left(k\right)=\frac{1}{n\left(k\right)}\underset{\left(x_{j},r_{j},{ŷ}_{j},y_{j}\right)\in D_{\text{pseudo},\text{val}}^{\left(k\right)}}{\sum }1\left[{ŷ}_{j}=y_{j}\right]. \end{array}$$ (11)

Here, Prec(*k*) serves as a practical proxy for pseudo-label quality, while ρ(*k*) captures the relative amount of additional supervision made available from the positive pool for downstream training.

To score pseudo-label quality, we do not directly use observed precision alone. Instead, we use a practical approximation motivated by symmetric label noise. Although the true error pattern in pseudo-labels is not necessarily symmetric, and the exact downstream trade-off between precision and sample count is generally unknown, symmetric-noise intuition provides a useful reference point for calibrating this trade-off. Under a symmetric label-noise rate η, the effective supervision signal is attenuated in proportion to 1 − 2η. If we use the observed precision Prec(*k*) as a proxy for the correctness rate and identify η = 1 − Prec(*k*), the signed effective signal is 2Prec(*k*)−1. Values above 0.5 indicate positively informative pseudo-labels, whereas values below 0.5 indicate anti-informative pseudo-labels. We therefore define the signed noise-aware quality proxy in Equation 12:


$$\begin{array}{l} k_{\text{noise}}\left(\text{Prec}\left(k\right)\right)=\text{sgn}\left(2\text{Prec}\left(k\right)-1\right)|2\text{Prec}\left(k\right)-1{|}^{2}. \end{array}$$ (12)

The squared magnitude follows the effective-signal interpretation under symmetric label noise: as the retained subset approaches random correctness, Prec(*k*) → 0.5, the usable signal should rapidly vanish. The sign term preserves the direction of the signal, assigning negative utility to anti-informative operating points with Prec(*k*) < 0.5. Thus, thresholds below chance are explicitly disfavored rather than being treated only by their distance from random correctness.

At the same time, thresholds with very small retained sets can appear deceptively strong when the validation set is limited, because a small number of favorable outcomes may produce an overly optimistic precision estimate. To reduce such overconfidence, we additionally incorporate a conservative finite-sample adjustment based on the Wilson lower bound of precision. For a candidate retention rate *k*, let *n*(*k*) be the number of retained validation examples and let Prec(*k*) be the observed precision defined in Equation 11. We then define the Wilson lower bound at *k* in Equation 13 as


$$\begin{array}{l} \text{LB}\left(k\right)=\frac{\text{Prec}\left(k\right)+\frac{c^{2}}{2n\left(k\right)}-c\sqrt{\frac{\text{Prec}\left(k\right)\left(1-\text{Prec}\left(k\right)\right)}{n\left(k\right)}+\frac{c^{2}}{4n{\left(k\right)}^{2}}}}{1+\frac{c^{2}}{n\left(k\right)}}, \end{array}$$ (13)

where *c* is the standard-normal critical value corresponding to the desired confidence level; in line with standard practice, we set *c* = 1.96, corresponding to a 95% confidence level. As given in Equation 13, LB(*k*) becomes more conservative when *n*(*k*) is small, making it better suited than a naive point estimate in limited-validation settings (NIST/SEMATECH, 2024). This choice is also aligned with prior work on confidence-aware pseudo-label selection, which emphasizes that high-confidence but incorrect pseudo-labels can amplify confirmation bias (Rizve et al., 2021).

Combining the finite-sample adjustment in Equation 13, the signed noise-aware quality proxy in Equation 12, and the retained ratio ρ(*k*), we define the score of retention rate *k* in Equation 14:


$$\begin{array}{l} \text{score}\left(k\right)=\text{LB}{\left(k\right)}^{1-\alpha }\cdot \phi_{\alpha }\left(k_{\text{noise}}\left(\text{Prec}\left(k\right)\right)\right)\cdot \rho {\left(k\right)}^{\beta }, \end{array}$$ (14)

where


$$\begin{array}{l} \phi_{\alpha }\left(u\right)=\text{sgn}\left(u\right)|u{|}^{\alpha }. \end{array}$$ (15)

Here, LB(*k*) is the Wilson lower bound defined in Equation 13, *k*_noise_(Prec(*k*)) is the signed noise-aware quality proxy defined in Equation 12, and ρ(*k*) is the retained positive-pool ratio defined in Equation 10. Using ρ(*k*) rather than the raw count *n*(*k*) keeps the quantity term on a bounded scale and makes the score less dependent on the absolute size of the verifier-validation positive pool. Because *k*_noise_ can be negative when the retained subset is anti-informative, we apply the exponent through the signed-power transform $\phi_{\alpha }\left(u\right)=\text{sgn}\left(u\right)|u{|}^{\alpha }$, rather than directly raising a negative value to a fractional power.

In practice, we search over candidate retention rates *k* that define the top-*k* threshold in Equation 7. We first evaluate candidates coarsely in 5% increments and then refine the search around promising regions in 1% increments. The final retention rate is chosen according to Equation 16:


$$\begin{array}{l} k^{*}=\arg\underset{k}{\max}\text{score}\left(k\right). \end{array}$$ (16)

The selected k^*^ determines the corresponding entropy cutoff $\tau_{k^{*}}$ in Equation 7, which in turn specifies the final pseudo-labeled subset $D_{\text{pseudo}}^{\left(k^{*}\right)}$ and the downstream training set $D_{\text{train}}^{\left(k^{*}\right)}$ in Equation 8. As defined in Equation 16, this gives a single validation-based rule for choosing the top-*k* operating point by jointly accounting for pseudo-label quality, retained ratio, and finite-sample confidence through the score in Equation 14. Equation 14 is intended as a practical validation-based criterion rather than a uniquely optimal utility function. Its role is to make the operating-point trade-off explicit, which is better aligned with minimal-label pseudo-label selection than relying on a fixed top-k rule.

### 3.3 Confidence-aware verifier training

The adaptive threshold-selection criterion in Section 3.2 works best when the verifier in Section 3.1 satisfies two conditions: it should be a strong correctness classifier, and its confidence should be well aligned with pseudo-label reliability. Entropy-based selection depends on both. If correctness prediction is weak, the candidate pool itself is noisy; if confidence is poorly aligned, entropy cannot reliably rank pseudo-label quality.

GRPO-style reinforcement learning is known to be effective for improving prediction correctness beyond supervised fine-tuning. However, correctness-oriented RL can also weaken the correspondence between entropy and correctness, making the verifier more overconfident and less suitable for entropy-based selection. To address this trade-off, we train the verifier *M*_ver_ in Section 3.1 with a CAVT-style reward.

Concretely, we optimize the verifier on the verifier-training set *D*_ver_ in Equation 1. For each verifier-training example (*x*_*i*_, *r*_*i*_, ŷ_*i*_, *z*_*i*_) ∈ *D*_ver_, the verifier produces the next-token distribution *p*_*i*_(*v*) defined in Equation 2. We then measure the verifier's predictive uncertainty using the token entropy of this distribution:


$$\begin{array}{l} H\left(p_{i}\right)=-\underset{v\in V}{\sum }p_{i}\left(v\right)\log p_{i}\left(v\right). \end{array}$$ (17)

As in Equation 17, lower token entropy corresponds to sharper verifier confidence.

Let ${\tilde{z}}_{i}\in \left\{0,1\right\}$ denote the verifier's sampled binary prediction for example *i*. We define the signed correctness term in Equation 18 as


$$s_{i}=\{\begin{array}{ll} +1, & \text{if }{\tilde{z}}_{i}=z_{i},\\ -1, & \text{otherwise,} \end{array}$$ (18)

where *z*_*i*_ is the gold correctness label introduced in Section 3.1. Using Equations 17, 18, we define the CAVT reward for verifier training in Equation 19 as


$$\begin{array}{l} R_{i}=s_{i}\log_{b}\left(\frac{1}{H\left(p_{i}\right)+\varepsilon }\right), \end{array}$$ (19)

where *b* is the logarithm base and ε>0 is a small constant for numerical stability.

Equations 18, 19 imply that low-entropy correct verifier predictions receive positive reward, while low-entropy incorrect predictions receive negative reward. In contrast, high-entropy predictions produce rewards with smaller magnitude. This design is well suited to our selection-oriented setting. The threshold-selection rule in Section 3.2 relies on the verifier not only to distinguish correct from incorrect pseudo-labels, but also to rank them by token entropy in a way that is useful for filtering. By encouraging *M*_ver_ to be confident mainly when it is correct, CAVT aims to improve the entropy–correctness correspondence and thereby support more reliable threshold selection.

In practice, we use this CAVT reward in Equation 19 within the same GRPO-based training framework as prior work, replacing the verifier-training objective in Equation 3 with a reinforcement-learning objective for *M*_ver_. In combination with adaptive threshold selection, this yields a verifier whose confidence is more useful for selecting high-utility pseudo-labels, leading to more effective semi-supervised reasoning under minimal labels.

## 4 Experiments

### 4.1 Experimental setup

#### 4.1.1 Task overview

We evaluate our method in a verifier-based semi-supervised reasoning setting, following prior work on learning from minimal labels through verifier-guided pseudo-label selection (Kato et al., 2026b). In this setting, the model first generates reasoning traces and final answers for a small labeled set, and a verifier is trained to judge whether each generated solution is correct. The trained verifier is then applied to a larger pool of unlabeled questions, and only pseudo-labeled samples judged to be sufficiently reliable are retained for downstream self-training. In this way, the system improves reasoning ability by generating candidate solutions, filtering them through a verifier, and reusing the selected high-confidence samples as additional supervision.

More concretely, given a small labeled set of question–answer pairs, the generator first produces reasoning traces and answers. These generations are compared with the gold answers to derive binary correctness labels for verifier training. After the verifier is trained, it is applied to candidate reasoning traces generated from unlabeled questions, and the selected pseudo-labeled traces are used to fine-tune the downstream reasoning model. Our setting follows the same overall semi-supervised framework as prior work, while revisiting both verifier training and threshold selection so that pseudo-label quality and quantity can be better balanced under minimal supervision.

For each dataset, backbone, and verifier-training condition, we run the full experimental pipeline once. The reported comparisons are based on this single-run setup.

#### 4.1.2 Datasets and data split

We used three mathematical reasoning subsets from the Verifiable Math Problems benchmark (PrimeIntellect, 2025): *orca_math, synthetic_math*, and *cn_k12*. We chose these datasets for several practical reasons. As mathematical reasoning tasks, they allow final-answer correctness to be determined automatically, which is important for verifier-based training and evaluation. They also require intermediate reasoning rather than superficial pattern matching, making them well suited to our study of reasoning-oriented semi-supervised learning. Compared with more widely used benchmarks such as GSM8K, these subsets are also less saturated in prior LLM training and evaluation, which may reduce the risk of contamination or data leakage. In addition, although all three datasets belong to verifiable mathematical reasoning, they span substantially different starting conditions for pseudo-label selection. In our setup, both the zero-shot downstream accuracy and the positive/negative ratio of candidate pseudo-labels vary widely across datasets and model backbones, indicating that the initial quality and density of potentially useful pseudo-labels are far from uniform. From the perspective of verifier-guided semi-supervised learning, this variation is important because it changes the difficulty of selecting a useful operating point under the quality–quantity trade-off.

For each dataset, we randomly construct the following split. We use 200 labeled examples for verifier training and 200 labeled examples for verifier validation. In addition, we sample 10,000 unlabeled examples for pseudo-label generation and semi-supervised downstream training. For downstream evaluation, we use 100 labeled examples as a validation set for early stopping and 500 labeled examples as a held-out test set. Thus, during the training term, the total number of labeled examples used per dataset is 500, and only 400 are allocated to verifier learning and threshold selection. This split is intentionally designed to keep labeled supervision as small as possible while still preserving a minimally stable basis for verifier training and validation-based threshold optimization. Under this setup, the verifier must be learned from very limited supervision, and the success of semi-supervised learning depends heavily on whether reliable pseudo-labels can be selected from the unlabeled pool.

#### 4.1.3 Prompting and verifier formulation

Following prior work on binary verifier training for truthfulness judgment, we formulate verifier learning as a binary decision problem. Each example is converted into an instruction-style prompt, and the verifier is trained to output one of two labels, 1 or 0, depending on whether the given solution is correct. For the mathematical datasets, we use the following template:

Let's think step by step. The mathematical question and its solution are given. If the solution is correct, return TRUE; otherwise, return FALSE.question:{question}solution:{solution}

This binary formulation is used for all verifier-training methods in our comparison. For supervised training, the model is optimized to predict the correct binary label. For reinforcement-learning-based methods, the same prompts and binary outputs are used, but the verifier is further optimized with different reward designs that affect the relation between correctness and confidence.

#### 4.1.4 Model backbones

We use Llama-3.2-3B-Instruct (Grattafiori et al., 2024) and Qwen-2.5-3B-Instruct (Qwen, 2024) as the base models. These are widely used open-source backbones, and they allow us to compare behavior across two different model families under a practically trainable setting.

#### 4.1.5 Training details

**Verifier training**. For SFT of the verifier, we follow the same setting as prior work (Kato et al., 2026b) on binary verifier training. We use the trl (Werra et al., 2020) library, batch size 64, learning rate 1 × 10^−6^, and warm-up ratio 0.05. Unless otherwise noted, the remaining optimization settings follow the default configuration of trl.

For reinforcement-learning-based verifier training, namely GRPO, EM-RL, and CAVT, we follow the same implementation scale as prior work (Kato et al., 2026a) and use Verl (Sheng et al., 2024). The number of rollouts is set to *N* = 4, the batch size to 128, and the learning rate to 1 × 10^−6^. We use KL regularization with coefficient β = 0.001, and all remaining RL-specific hyperparameters, including clipping and advantage-normalization options, follow the default settings of Verl.

Our early-stopping rule for verifier training is selected to match the downstream goal of pseudo-label selection. Instead of stopping solely by verifier classification quality, we select the checkpoint that maximizes the validation score used for threshold determination. This is because the role of the verifier in our pipeline is not only to classify correctness, but to support reliable pseudo-label selection under a precision–coverage trade-off.

**Downstream training**. After pseudo-label selection, the downstream generator is trained on the union of the original labeled set and the selected pseudo-labeled samples. For this stage, we follow the same semi-supervised self-training setup as prior work on verifier-guided reasoning expansion. The downstream model is fine-tuned with standard supervised training on the selected reasoning traces, and early stopping is determined by downstream-task accuracy on the downstream validation set of 100 labeled examples.

#### 4.1.6 Baselines

We evaluate our method in two steps. First, we compare the conventional fixed-threshold strategy with adaptive threshold selection in order to test whether threshold choice itself matters for verifier-based semi-supervised learning. In this comparison, we focus on the SFT verifier as the common reference, and in the ablation study we additionally report CAVT under both thresholding strategies. Second, after establishing the importance of adaptive thresholding, we compare different verifier-training objectives under the adaptive setting—SFT, GRPO, EM-RL, and CAVT—to examine which type of verifier is most suitable for selecting a large number of trustworthy pseudo-labeled samples.

**Conventional fixed-threshold baseline: SFT + fixed top-10%** (Kato et al., 2026b). This is the direct baseline from prior verifier-based semi-supervised learning. A verifier is trained with standard SFT, and pseudo-labeled samples are selected by retaining the top 10% most confident examples according to entropy. This setting corresponds to the original fixed-threshold selection strategy used in prior work and serves as the main reference point for evaluating whether adaptive threshold selection provides additional benefit.

**Adaptive-threshold methods (proposed)**. Under adaptive threshold selection, we compare the following four verifier-training methods.

**SFT:** This condition uses a verifier trained with standard SFT and applies our validation-based threshold selection instead of the fixed top-10% rule. It isolates the effect of adaptive thresholding without changing the verifier training objective.

**GRPO:** This is a correctness-based reinforcement-learning baseline. It optimizes the verifier with standard binary rewards and a PPO-style group-relative objective. This baseline tests whether a verifier trained to improve correctness under RL can also support effective pseudo-label selection when thresholding is adapted on validation data. Prior work has shown that GRPO can improve predictive performance, but also that it may weaken the correspondence between entropy and correctness, which is problematic when entropy is used for sample selection.

**EM-RL** (Agarwal et al., 2025): This is an entropy-minimization reinforcement-learning baseline. Rather than rewarding correctness directly, it encourages the model to reduce predictive entropy, with the intuition that sharper distributions may yield stronger confidence separation. This makes EM-RL relevant to our setting, where the goal is to retain as many reliable pseudo-labeled samples as possible. However, because its reward does not use ground-truth correctness labels, it can also reinforce confidently wrong predictions and lead to unstable verifier behavior. Prior work has noted this trade-off: entropy minimization may sometimes improve confidence separation, but it can do so at the cost of predictive reliability. Testing EM-RL therefore helps assess whether confidence sharpening without correctness-aware supervision is sufficient for robust pseudo-label selection.

**CAVT:** This is our main method. It trains the verifier with a confidence-aware reward that encourages low entropy when predictions are correct and discourages low entropy when predictions are incorrect. Its intended advantage in the present setting is especially direct: if the goal is to retain as many reliable pseudo-labeled samples as possible, then the best verifier is not simply the one with the highest classification accuracy, but the one whose confidence ranking most faithfully reflects correctness. CAVT is therefore designed to make entropy more usable as a pseudo-label selection signal under adaptive thresholding.

Overall, these baselines allow us to compare the conventional fixed-threshold strategy against adaptive thresholding, and to evaluate which verifier-training objective is most compatible with the goal of selecting the largest possible set of trustworthy pseudo-labels.

#### 4.1.7 Computational cost

The adaptive threshold search is performed only on the verifier-validation set after verifier training. It requires only sorting candidates by entropy and evaluating a finite grid of retention rates, so its additional computational cost is negligible compared with verifier training and downstream fine-tuning. CAVT uses the same GRPO-based training framework and rollout scale as the RL-based verifier-training baselines, so its cost is comparable to GRPO and EM-RL in our experimental setup.

#### 4.1.8 Other hyper parameters

For the hyperparameters in Equation 14, we keep the Wilson contribution modest by setting 1−α = 0.1 (i.e., α = 0.9), so that the score is driven primarily by the noise-aware quality term while still incorporating a conservative finite-sample correction. We then set β = 0.9, so that the two main components of the trade-off—noise-aware pseudo-label quality and retained sample count—are weighted on a comparable scale. These values are not intended as a theoretically optimal derivation; rather, they provide a practical calibration for selecting an operating point when the exact downstream utility function is unknown. For the hyperparameters in Equation 19, we set *b* = 10 and ϵ = 1*e*−6 as in Kato et al. (2026a).

#### 4.1.9 Evaluation metrics

Our primary evaluation target is the downstream-task accuracy after semi-supervised training. Since the final goal of the framework is to improve reasoning performance on the downstream task, downstream accuracy is the most important metric and serves as the main basis for model comparison.

As auxiliary analyses, we also report several verifier-side and selection-side metrics. First, we evaluate pseudo-label judgment quality using precision, recall, and F1. Second, we report AUPC, the area under the precision–coverage curve, which measures how well a verifier maintains precision as more samples are retained under confidence-based selection. This metric is useful in our setting because the goal is not merely to identify a few very clean samples, but to retain as many reliable samples as possible. Finally, for the threshold actually selected in each condition, we report the resulting precision at that operating point (Prec.@*k*) and the number of retained samples. Together, these metrics help clarify not only whether a verifier is accurate as a binary classifier, but also whether it is useful as a sample-selection mechanism for semi-supervised learning.

### 4.2 Qualitative sanity check of the threshold-selection rule

**Figure 1:**
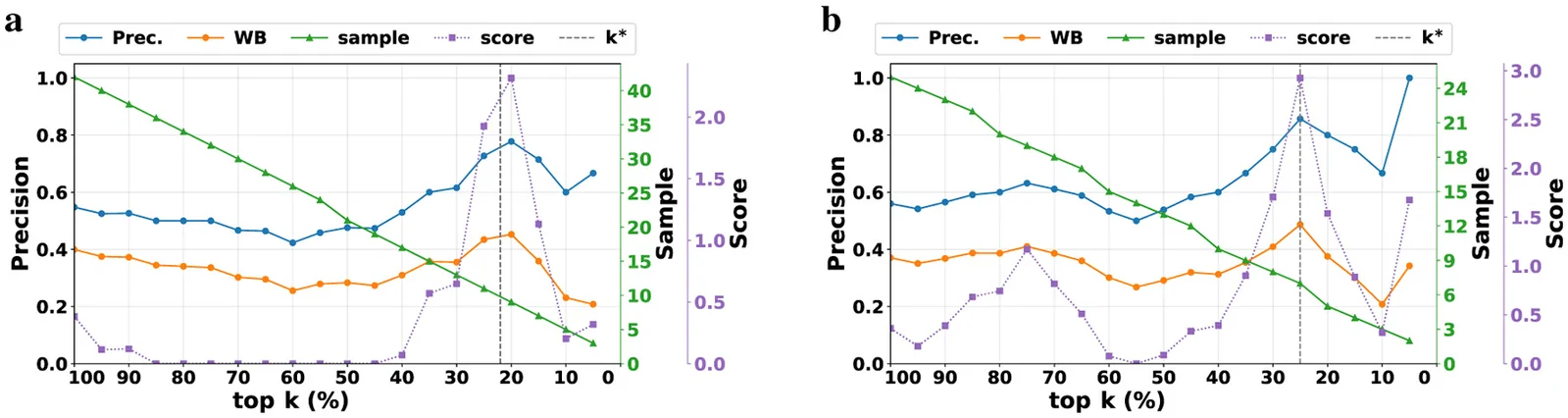
Examples of precision–coverage-related curves used for adaptive threshold selection. The horizontal axis shows the retained top-k percentage, and the plotted lines indicate observed precision (Prec.), Wilson-lower-bound precision (WB), retained sample count (sample), and the resulting threshold-selection score (score). The vertical dashed line indicates the selected threshold k^*^. **(a)** Case where the highest-confidence region is not the highest-precision region. **(b)** Case where the extreme high-confidence region appears strong but is sample-limited.

We begin with a qualitative sanity check of whether the proposed threshold-selection rule behaves reasonably, by visualizing representative precision–coverage patterns and the resulting score in Figure 1. In both figures, the horizontal axis shows the retained top-k percentage. Moving to the right corresponds to retaining fewer samples and applying a more restrictive confidence threshold. The blue line (Prec.) shows the observed precision, the orange line (WB) shows the Wilson lower bound of precision, the green line (sample) shows the sample-count term, the purple line (score) shows the resulting threshold-selection score, and the vertical dashed line (k^*^) indicates the selected threshold.

Figure 1a illustrates a case where the highest-confidence region does not coincide with the highest-precision region. In such cases, a fixed top-10% rule can be suboptimal, because the most confident tail is not necessarily the most useful operating point for downstream training. By contrast, Figure 1b shows a case where precision appears to improve in the extreme high-confidence region, making fixed top-10% filtering look plausible at first glance. However, once the retained sample count becomes very small, such high precision may be unreliable as an operating-point estimate. Incorporating the Wilson lower bound helps avoid over-trusting these extreme regions and instead favors thresholds that better balance pseudo-label quality and retained sample count. Overall, these examples suggest that the proposed score behaves qualitatively in line with the intended quality–quantity trade-off, rather than simply selecting the most confident tail.

### 4.3 Overall results

Tables 1, 2 summarize the main results for Llama3.2-3B-Instruct and Qwen2.5-3B-Instruct, respectively. We report downstream accuracy with 95% Wilson confidence intervals, together with verifier-side and selection-side diagnostics: precision, recall, F1, AUPC, the selected top-k retention rate, its bootstrap interquartile range (IQR, computed from 1,000 resamples), precision at the selected operating point (Prec.@k), and the retained pseudo-label sample count. The Wilson confidence intervals for downstream accuracy summarize uncertainty due to the finite held-out test set, and the bootstrap IQRs for selected thresholds summarize sensitivity to resampling the verifier-validation set. Both quantities are single-run uncertainty summaries computed from the reported experimental run. All metrics except the sample count are reported in percentage form. For comparison, we additionally show the zero-shot downstream accuracy of the base model. In the tables, SFT (top-10) denotes the conventional supervised verifier with a fixed top-10% threshold (Kato et al., 2026b), while all other methods use the proposed validation-based threshold selection.

**Table 1:** Results for Llama3.2-3B-Instruct.

Dataset	Method	Prec.	Rec.	F1	AUPC	Acc.	k	IQR	Prec@k	Sample
orca_math	zero-shot	—	—	—	—	29.2 ± 4.1	—	—	—	
	SFT (top-10)	76.8	73.5	75.1	85.0	42.8 ± 4.4	10	—	90.7	269
	SFT	76.8	73.5	75.1	85.0	**48.8** **±4.4**	79	73–85	81.5	2,121
	GRPO	67.5	**90.8**	77.4	81.1	48.4 ± 4.4	53	48–59	82.4	1,999
	EM-RL	**86.9**	42.8	57.3	**91.5**	45.8 ± 4.4	100	98–100	**86.9**	1,380
	CAVT	78.5	77.0	**77.8**	86.5	48.6 ± 4.4	70	63–76	84.7	1,924
synthetic_math	zero-shot	—	—	—	—	21.4 ± 3.8	—	—	—	
	SFT (top-10)	63.2	62.5	62.8	69.7	21.4 ± 3.8	10	—	74.4	199
	SFT	63.2	62.5	62.8	69.7	24.4 ± 4.0	45	38–53	71.4	894
	GRPO	66.0	59.3	62.4	72.0	25.8 ±4.0	54	41–61	73.3	974
	EM-RL	62.7	**64.6**	**63.6**	68.5	23.6 ± 3.9	22	18–32	**74.3**	455
	CAVT	**71.2**	53.8	61.3	**75.2**	**27.4** **±4.1**	80	69–84	73.3	1,215
cn_k12	zero-shot	—	—	—	—	12.0 ± 3.1	—	—	—	
	SFT (top-10)	46.4	39.1	42.4	49.6	13.4 ± 3.3	10	—	53.2	94
	SFT	46.4	39.1	42.4	49.6	13.6 ± 3.3	11	10–28	55.3	103
	GRPO	**48.5**	**53.8**	**51.0**	53.3	14.6 ± 3.4	25	20–39	56.5	308
	EM-RL	46.7	15.8	23.6	49.0	13.6 ± 3.3	32	21–47	54.2	120
	CAVT	48.0	51.5	49.7	**54.1**	**17.4** **±3.6**	45	39–53	**57.1**	536
Average	zero-shot	—	—	—	—	20.9 ± 2.2	—			
	SFT (top-10)	62.1	58.4	60.1	68.1	25.9 ± 2.2				
	SFT	62.1	58.4	60.1	68.1	28.9 ± 2.3				
	GRPO	60.6	**68.0**	**63.6**	68.8	29.6 ± 2.4				
	EM-RL	65.4	41.0	48.2	69.7	27.7 ± 2.3				
	CAVT	**65.9**	60.8	62.9	**71.9**	**31.1** **±2.4**				

Acc. is reported with 95% Wilson confidence intervals. Gray rows indicate the baseline conditions [zero-shot and fixed-threshold SFT (top-10)]; all other methods use the proposed validation-based threshold selection. Boldface indicates the best value for performance and verifier-side diagnostic metrics.

**Table 2:** Results for Qwen2.5-3B-Instruct.

Dataset	Method	Prec.	Rec.	F1	AUPC	Acc.	k	IQR	Prec@k	Sample
orca_math	zero-shot	—	—	—	—	49.2 ± 4.4	—	—	—	
	SFT (top-10)	79.5	74.6	77.0	87.1	47.8 ± 4.4	10	—	92.8	442
	SFT	79.5	74.6	77.0	87.1	51.4 ± 4.4	67	61–75	85.7	2,961
	GRPO	73.7	**88.2**	80.3	80.3	53.6 ± 4.4	79	69–83	80.0	4,451
	EM-RL	**81.6**	68.4	74.4	**88.7**	51.6 ± 4.4	76	69–83	**85.9**	2,998
	CAVT	79.5	86.4	**82.8**	82.5	**53.8** **±4.4**	94	91–96	81.8	4,810
synthetic_math	zero-shot	—	—	—	—	24.6 ± 4.0	—	—	—	
	SFT (top-10)	67.6	74.4	70.8	80.1	25.0 ± 4.0	10	—	92.9	254
	SFT	67.6	74.4	70.8	80.1	26.2 ± 4.0	33	23–45	85.3	838
	GRPO	**76.0**	64.7	69.9	**84.3**	26.4 ± 4.0	52	39–59	87.0	1,023
	EM-RL	62.4	83.7	71.5	78.0	26.2 ± 4.0	28	22–42	**87.1**	868
	CAVT	58.9	**92.5**	**72.0**	73.2	**26.8** **±4.0**	57	50–63	74.2	2,067
cn_k12	zero-shot	—	—	—	—	18.0 ± 3.6	—	—	—	
	SFT (top-10)	57.9	67.2	62.2	65.7	19.0 ± 3.7	10	—	71.0	200
	SFT	57.9	67.2	62.2	65.7	**21.2** **±3.8**	57	49–62	66.5	1,135
	GRPO	**58.7**	48.2	52.9	64.9	20.8 ± 3.8	67	59–71	67.3	946
	EM-RL	56.0	76.1	64.5	66.0	21.0 ± 3.8	41	35–50	70.4	957
	CAVT	56.4	**77.6**	**65.3**	**66.6**	21.0 ± 3.8	45	40–51	**70.5**	1,064
Average	zero-shot	—	—	—	—	30.6 ± 2.4	—			
	SFT (top-10)	68.3	72.1	70.0	**77.6**	30.6 ± 2.4				
	SFT	68.3	72.1	70.0	**77.6**	32.9 ± 2.4				
	GRPO	**69.5**	67.0	67.7	76.5	33.6 ± 2.4				
	EM-RL	66.7	76.1	70.1	**77.6**	32.9 ± 2.4				
	CAVT	64.9	**85.5**	**73.4**	74.1	**33.9** **±2.4**				

All results are presented in the same manner in the Table 1. Boldface indicates the best value for each metric within each dataset. Gray rows indicate the baseline conditions (zero-shot and fixed-threshold SFT (top-10)); all other methods use the proposed validation-based threshold selection.

#### 4.3.1 Fixed top-10% vs. adaptive thresholding for SFT

Before comparing verifier-training methods, we first examine whether the threshold-selection strategy itself matters by comparing SFT (top-10) with SFT. This isolates the effect of replacing the conventional fixed top-10% rule with the proposed adaptive threshold selection while keeping verifier training unchanged.

Across both backbones, adaptive thresholding tends to improve downstream accuracy over fixed top-10% filtering. These results suggest that adaptive thresholding can provide a useful alternative to fixing the threshold at top 10%, especially when the appropriate quality–coverage balance varies across datasets.

In particular, on Llama3.2-3B-Instruct for orca_math, adaptive SFT selects a broader subset while maintaining Prec.@k above 80%, whereas SFT(top-10) retains only the most conservative tail. Similarly, on Qwen2.5-3B-Instruct for orca_math, SFT(top-10) falls below the zero-shot baseline, whereas adaptive SFT improves over both. These observations suggest that overly conservative filtering can leave too little data for effective self-training. Consistent with this interpretation, the bootstrap IQRs show that the selected thresholds are generally concentrated around the original selected k, although variability becomes larger in some low-resource settings such as Llama3.2-3B-Instruct on cn_k12.

#### 4.3.2 Comparison across verifier-training methods

Having established that adaptive thresholding is preferable to the conventional fixed top-10% rule, we next compare different verifier-training objectives under the adaptive setting: SFT, GRPO, EM-RL, and CAVT.

For Llama3.2-3B-Instruct, CAVT gives the best average downstream accuracy among the adaptive methods. The gains are particularly visible on synthetic_math and cn_k12, where CAVT improves over both SFT and the RL-based baselines. The verifier-side diagnostics are also broadly consistent with these downstream results: CAVT performs well on ranking-oriented metrics, including AUPC, while maintaining competitive precision and recall. This suggests that, in the Llama setting, confidence-aware verifier training can make entropy scores more useful for adaptive pseudo-label selection.

For Qwen2.5-3B-Instruct, the margins among adaptive methods are smaller than in the Llama results, but CAVT still gives the best average downstream accuracy and remains competitive with GRPO across datasets. The verifier-side diagnostics are more mixed. CAVT achieves the best average F1 and recall, but its average AUPC is lowered mainly by synthetic_math, where it moves to a substantially higher-recall and lower-precision regime. When precision and recall differ this strongly across methods, AUPC is still informative but becomes harder to interpret as a standalone indicator of downstream utility. Indeed, outside synthetic_math, CAVT shows a more favorable verifier-side profile, with strong F1 and competitive or higher AUPC. These results suggest that, in the Qwen setting, CAVT is a competitive alternative to GRPO under adaptive thresholding, with a modest but favorable downstream tendency.

Taken together, these results suggest that the effectiveness of pseudo-label selection depends on both verifier behavior and threshold choice. More broadly, pseudo-label utility is not determined by a single verifier metric in isolation, but by how well the selected operating point balances pseudo-label reliability and retained sample count for downstream self-training.

As an additional important point, although our evaluation is restricted to verifiable mathematical reasoning, the same tendency is observed across substantially different starting conditions, including wide variation in zero-shot accuracy and the positive/negative balance of candidate pseudo-labels.

### 4.4 Ablation study

Table 3 presents an ablation study designed to isolate the contribution of adaptive threshold selection from that of CAVT-based verifier training. While Tables 1, 2 report a broader set of verifier-side and downstream metrics to give an overall comparison across methods, the key question in the present ablation is more specific: whether the gain comes from threshold selection, from CAVT itself, or from their combination. For this purpose, Table 3 focuses on the most directly relevant quantities for that comparison—downstream accuracy, the selected top-*k*, precision at the selected operating point (Prec.@k), and the number of retained pseudo-labeled samples. We therefore organize the comparison along two axes: fixed top-10% vs. adaptive thresholding, and SFT vs. CAVT. This lets us ask two questions separately: whether CAVT is effective by itself under the conventional fixed-threshold setting, and whether its effect becomes larger when combined with the proposed threshold-selection rule.

**Table 3:** Results for ablation study.

Dataset	Method	Llama-3.2-3B-Instruct				Qwen 2.5-3B-Instruct			
		Acc.	k	Prec@k	Sample	Acc.	k	Prec@k	Sample
orca_math	zero-shot	29.2 ± 4.1	—	—	—	49.2 ± 4.4	—	—	—
	SFT (top-10)	42.8 ± 4.4	10.0	90.7	269	47.8 ± 4.4	10.0	**92.8**	442
	CAVT (top-10)	42.8 ± 4.4	10.0	**92.0**	275	47.2 ± 4.4	10.0	82.6	512
	SFT	**48.8** **±4.4**	79.0	81.5	2,121	51.4 ± 4.4	67.0	85.7	2,961
	CAVT	48.6 ± 4.4	70.0	84.7	1,924	**53.8** **±4.4**	94.0	81.8	4,810
synthetic_math	zero-shot	21.4 ± 3.8	—	—	—	24.6 ± 4.0	—	—	—
	SFT (top-10)	21.4 ± 3.8	10.0	74.4	199	25.0 ± 4.0	10.0	**92.9**	254
	CAVT (top-10)	21.8 ± 3.8	10.0	**80.9**	152	25.4 ± 4.0	10.0	85.1	363
	SFT	24.4 ± 4.0	45.0	71.4	894	26.2 ± 4.0	33.0	85.3	838
	CAVT	**27.4** **±4.1**	80.0	73.3	1,215	**26.8** **±4.0**	57.0	74.2	2,067
cn_k12	zero-shot	12.0 ± 3.1	—	—	—	18.0 ± 3.6	—	—	—
	SFT (top-10)	13.4 ± 3.3	10.0	53.2	94	19.0 ± 3.7	10.0	**71.0**	200
	CAVT (top-10)	13.4 ± 3.3	10.0	54.6	119	19.0 ± 3.7	10.0	68.8	237
	SFT	13.6 ± 3.3	11.0	55.3	103	**21.2** **±3.8**	57.0	66.5	1,135
	CAVT	**17.4** **±3.6**	45.0	**57.1**	**536**	21.0 ± 3.8	45.0	70.5	1,064

Boldface indicates the best downstream accuracy (Acc.) for each dataset and backbone.

A clear overall pattern is that applying CAVT under fixed top-10% selection yields only very limited benefit. For Llama3.2-3B-Instruct, CAVT (top-10) slightly improves Prec.@*k* over SFT (top-10) on *orca_math* and *synthetic_math*, but the downstream accuracy is unchanged or nearly unchanged. In other words, even when the fixed top-10% subset has relatively high precision, this alone does not translate into meaningful downstream gains. For Qwen2.5-3B-Instruct, the picture is even less favorable to fixed-threshold CAVT: Prec.@*k* is consistently lower for CAVT (top-10) than for SFT (top-10), and on *orca_math* the downstream accuracy also drops from 47.8 to 47.2%, falling further below the zero-shot baseline of 49.2%.

By contrast, once CAVT is combined with adaptive thresholding, the improvement in downstream accuracy becomes much larger. For Llama3.2-3B-Instruct, CAVT improves over CAVT (top-10) from 42.8 to 48.6% on *orca_math*, from 21.8 to 27.4% on *synthetic_math*, and from 13.4 to 17.4% on *cn_k12*. For Qwen2.5-3B-Instruct, the corresponding gains are from 47.2 to 53.8%, from 25.4 to 26.8%, and from 19.0 to 21.0%. This shows that the effect of CAVT is not well realized under a fixed top-10% policy, but becomes much more visible when the threshold is selected adaptively.

The verifier-side statistics are also consistent with this interpretation. Under fixed top-10% selection, CAVT does not consistently improve precision at the selected operating point, especially for Qwen. Under adaptive thresholding, however, the gap in Prec.@*k* becomes smaller, and in some cases CAVT overtakes SFT. For example, on Llama3.2-3B-Instruct, CAVT exceeds SFT in Prec.@*k* on all three datasets, while on Qwen2.5-3B-Instruct the difference narrows substantially and CAVT remains competitive even when it selects many more samples. This suggests that CAVT is most useful when the thresholding rule can exploit the confidence structure it induces, rather than forcing all methods into the same narrow top-10% tail.

Taken together, these results indicate that the main effect does not come from CAVT alone. Rather, an important factor is the synergy between the proposed threshold-selection rule, which explicitly balances precision and retained sample count, and CAVT, whose confidence-aware training objective is better aligned with that selection principle. These results suggest that the observed gains are associated with the combination of adaptive threshold selection and CAVT, rather than with either component alone under the tested conditions.

## 5 Conclusion

This paper studied semi-supervised improvement of LLM reasoning under minimal-label conditions, focusing on verifier-based pseudo-label selection. We argued that the key challenge in this setting is not only how to train a verifier, but also how to select pseudo-labeled samples for downstream self-training. To address this, we proposed an integrated design that combines adaptive threshold selection with confidence-aware verifier training. The central idea is that pseudo-label selection should be treated as an explicit quality–quantity trade-off, and that entropy is useful for this purpose only when verifier confidence is well aligned with correctness.

Experiments on three verifiable math reasoning datasets with Llama-3.2-3B-Instruct and Qwen-2.5-3B-Instruct showed that the proposed approach improves downstream reasoning accuracy under minimal supervision. The results further showed that fixed top-10% filtering is often suboptimal, and that confidence-aware verifier training is most effective when combined with adaptive thresholding rather than used in isolation. Overall, our findings suggest that jointly designing threshold selection and verifier confidence leads to more useful pseudo-labeled subsets and more reliable downstream improvement in minimal-label verifier-guided reasoning.

## 6 Limitations

This study has several limitations.

First, our evaluation is limited to verifiable mathematical reasoning and a small set of model backbones. Although this setting is suitable for controlled pseudo-label selection, the present evidence does not establish that the same operating-point behavior will hold unchanged across broader reasoning tasks, open-ended generation settings, or more diverse model families.

Second, the experiments are based on a single run for each dataset, backbone, and verifier-training condition. Although we report Wilson confidence intervals for downstream accuracy and bootstrap IQRs for threshold sensitivity, these estimates do not capture full pipeline variability. In particular, they do not account for variation across verifier-training random seeds, data splits, threshold-selection runs, or downstream fine-tuning runs. Therefore, the empirical results should be interpreted as evidence from the evaluated single-run setup rather than as strong evidence of reliable superiority across repeated runs. A more complete assessment of robustness would require repeating the full pipeline across multiple seeds and data splits, which we leave for future work.

Third, our analysis is based on answer-level correctness labels and therefore does not fully characterize reasoning-trace quality or selection bias. This follows a common practical assumption in scalable reasoning self-training and verifier-guided pseudo-label selection, where generated traces with correct final answers are treated as usable supervision. However, final-answer correctness is not a complete proxy for trace utility, since incorrect or verifier-rejected traces may still contain useful local reasoning steps, contrastive information, or correction signals. Moreover, while we report selection-side diagnostics such as precision, recall, F1, AUPC, selected top-*k*, Prec.@*k*, and retained sample count, these metrics do not fully separate the effects of trace quality and problem difficulty. Such an analysis would require problem-level difficulty annotations, trace-level utility labels, or additional difficulty estimation, which are not available in the current benchmark setting. We leave these directions to future work.

Fourth, the proposed threshold score is a practical heuristic criterion rather than a theoretically optimal utility function. Our claim is therefore not that Equation 14 is uniquely correct, but that explicitly modeling the quality–quantity trade-off is more suitable for minimal-label pseudo-label selection than relying on a fixed threshold. A natural future direction is to move toward a more end-to-end formulation, in which verifier training and sample selection are guided more directly by downstream-task improvement rather than by a manually specified threshold score.

Fifth, we did not conduct a full sensitivity analysis over the score components and hyperparameters in Equation 14. Such an analysis would require repeating the full verifier-training, threshold-selection, downstream fine-tuning, and evaluation pipeline across multiple scoring variants, and we leave systematic component-level sensitivity analysis to future work.

Finally, our experiments use a single-round self-training pipeline. It remains an open question how the present gains would extend under iterative self-training, where better pseudo-label selection in one round could improve the candidate pool for subsequent rounds and potentially amplify the overall effect.

## Data Availability

The original contributions presented in the study are included in the article/supplementary material, further inquiries can be directed to the corresponding author.
